# Identification of *Theileria* Species in Sheep and Vector Ticks Using PCR Method in Zabol, Eastern Iran

**Published:** 2019-03-30

**Authors:** Fateme Zarei, Maryam Ganjali, Reza Nabavi

**Affiliations:** Department of Parasitology, Faculty of Veterinary Medicine, University of Zabol, Zabol, Iran

**Keywords:** *Theileria*, PCR, Iran

## Abstract

**Background:**

*Theileria* is a protozoal parasite that belongs to the phylum Apicomplexa. Theileriosis is an important tick-borne disease caused by various species of *Theileria*. Among these species, *T. lestoquardi* (*T. hirci*) is highly pathogenic, while other species such as *T. ovis* make Subclinical and mild infections in small ruminant. Therefore, the precise identification of the species and the vector ticks are very essential for epidemiological studies and the design of control programs.

**Methods:**

This research was conducted with the aim of molecular study to identify *Theileria* species and vectors in Zabol, eastern Iran in 2015. The presence of *Theileria* in 80 blood samples and vector ticks was evaluated using PCR method.

**Results:**

Of 80 blood samples, PCR analysis showed that 50 samples (62.5%) were infected with *Theileria.* The evaluation of the first phase PCR with Nested PCR showed that infections with *Theileria ovis* and *Theileria lestoquardi* were 67.45% and 32.55% cases respectively. Overall, 110 ticks (78 males and 32 females) were collected and generally two genera and six species including *Rhipicephalus bursa* (9.1%), *Rh. sanguineus* (29.1%), *Rh. turanicus* (10.9%) *Hyalomma asiaticum asiaticum* (23.63%), *Hy. excavatum* (10.9%), *Hy. anatolicum* (16.37%) were detected. After evaluating ticks infection by PCR method, three species of *Rh. turanicus*, *Rh. sanguineus* and *Hy. asiaticum asiaticum*, were infected.

**Conclusion:**

*Theileria ovis* has a high prevalence among the sheep of zabol and *Hy. asiaticum asiaticum*, *Rh. sanguineus* and *Rh. turanicus* may be the main vectors of *Theileria* species in this area.

## Introduction

Theileriosis is a tick-borne disease that infects domestic and wild ruminants in tropical and subtropical parts of the world, it causes serious problem for livestock husbandry in many parts of Iran (1). Two species of *Theileria ovis* and *Theileria lestoquardi* are the main causative of ovine Theileriosis in Iran. *Theileria lestoquardi* (= *T*. *hirci*) causes high mortality in sheep and goats in south and southeast of Iran. *Theileria ovis* is widely distributed in the country according to clinical and morphological observations (2). The ticks of the genera *Hyalomma* and *Rhipicephalus* have been reported in four geographic regions of Iran (3, 4). Ticks should be evaluated as *Theileria* vector in each region. Theileriosis not only directly lead to reduced production and mortality, but the cost of eradication of the ticks is added to the expense of the animal husbandry. This disease is also important in Iran, and it may be possible in the appropriate seasons to act as the main cause of the visit of livestock to veterinary clinics (5).

To classify and identify many haemopara-site ssuch as *Theileria*, molecular methods having high sensitivity and specificity compared to microscopic and serological tests (6, 7).

In this study, we tried to use the Nested-PCR method, which is a sensitive and specific method for identification of *Theileria* spp. in sheep and vector ticks in Zabol, eastern Iran. The results of the study will be effective in applying disease treatment and appropriate control measures.

## Materials and Methods

**Fig. 1.**
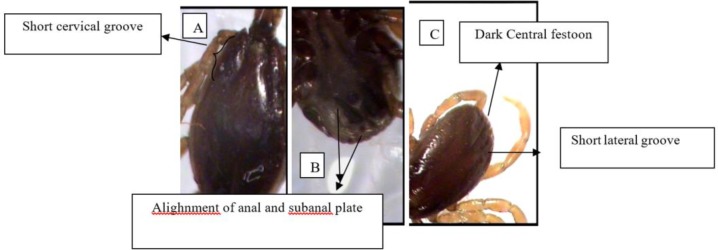
*Hyalomma anatolicum* male, (A) Dorsal view: cervical field depression is apparent (short cervical groove), (B) ventral view: subanal plates alignment is with the adanal plates (C) dorsal view: lateral grooves are short and central festoon is dark colored.

**Fig. 2.**
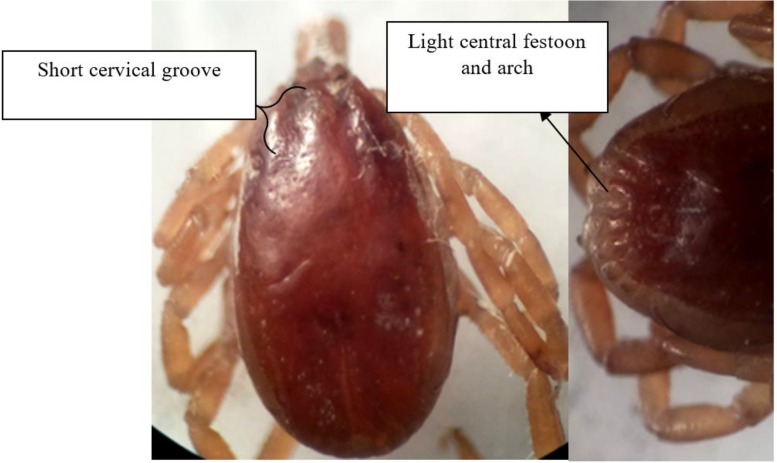
*Hyalomma excavatum* male, dorsal view: cervical field depression is apparent (short cervical groove), Lateral grooves are short, central festoon is light colored, paracentral festoons are fused. Ventral view: Subanal plates are in alignment with adanal plate

**Fig. 3.**
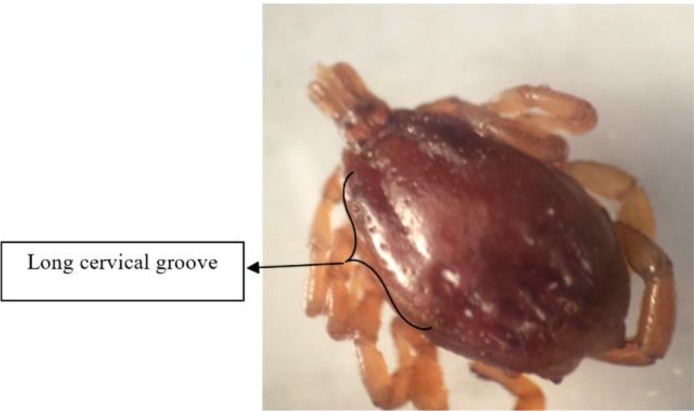
*Hyalomm asiaticum asiaticum* male, dorsal view: cervical grooves are long, other features include: lateral grooves are short, central festoon is triangular, Spiracles are long. Ventral view: Subanal plates are in alignment with adanal plate.

**Fig. 4.**
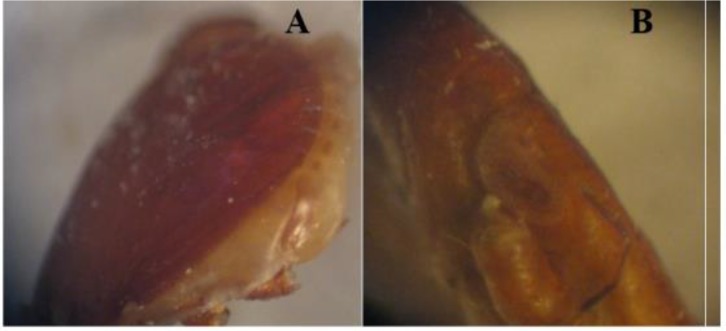
Spiracle of *Rhipicephalus turanicus* (A): male, (B): Female.

**Fig. 5.**
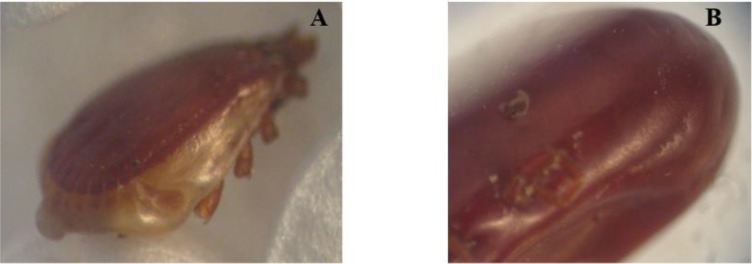
Spiracle of *Rhipicephalu sanguineus* (A): male, (B): Female

**Fig. 6.**
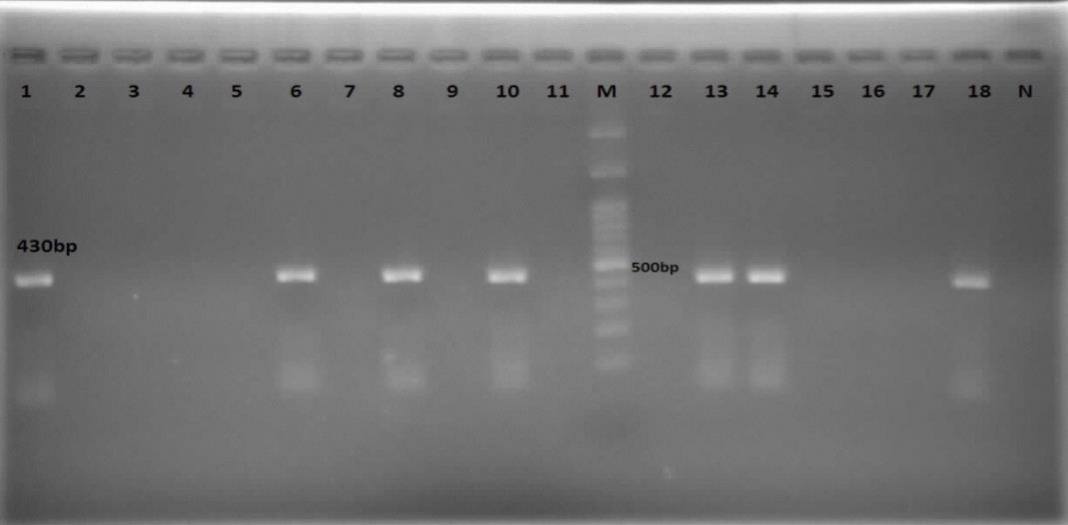
Agarose gel electrophoresis of PCR products related to extracted DNA from blood samples (band number: 1–11) and tick salivary glands (band number: 12–18). M 100bp ladder DNA marker; N negative control

Sistan and Baluchistan is located in southeast of Iran. Zabol has a desert climate and is located in the north of the province. During the spring and summer of 2015, 80 sheep were randomly selected from different areas of Zabol and were clinically examined. Blood samples were collected into tubes containing AL severs, solution (Citric Acid 0.55gr +Sodium Citrate 8gr +Dextrose 20.5gr +Sodium Chloride 4.2gr +D.W 1Lit). In the next stage, 110 ticks were removed from the ear and around the head, under the tail, anus and around the breast in females and scrotum in males. The ticks removed from each sheep were individually stored in labeled tube containing 70% alcohol and 5% glycerin. In order to identify ticks (Fig. 1–5), they were washed with sterilized water and were dried on sterilized filter paper and determined by the Walker identification key (8). Separation of tick salivary glands was performed according to Purnell method (9). After removing the tick scutum, salivary glands were homogenized in PBS (pH: 7.2), then centrifuged at 5,000rpm at 4 °C for 5min. The blood and ticks sample were used for DNA extraction using DNA Isolation kit (MBST, Iran) according to the kit instructions. The PCR reaction was then performed using primers with the sequence of TBF2: 5′cacagggaggtagtgacaag3′ and TBR2: 5′aagaatttcacctatgacag3′ (10, 11). DNA replication was performed using primers designed from flanking part of hyper-variable region of18S rRNA. The PCR product in all *Theileria* species is 426bp (Fig. 6). In order to identify the *Theileria* species, all blood samples were undergone the Semi-Nested PCR test with Specific inner primers derived from the 18S rRNA gene. In the second step, the nested inner primers were used for detection of *T. lestoquardi*, including TBR2: 5′-AGAATTTCACCTATGACAG-3′ and TL3: 5′-ATTGCTTGTGTCCCTCCG-3′ and the primers were used to detect *T. ovis* TBR2: 5′- AGAATTTCACCTATGACAG-3′ and TO4: 5′- TTGCTTTTGCTCCTTTA CGAG-3′ (10).

The PCR was performed on 50μl reaction volumes including, 4μl extracted DNA, 25μl Taq DNA polymerase 2× Master Mix (Pishgam Company, Iran), 3pmol of each primer and sterile distilled water up to 50ml in automated Thermocycler with the following program: 5min incubation at 95 °C to denature double strand DNA, 38 cycles of 45sec at 95 °C, 45sec at 56 °C (annealing step), 45sec at 72 °C and this was followed by final extension step at 72 °C for 10min.

**Fig. 7.**
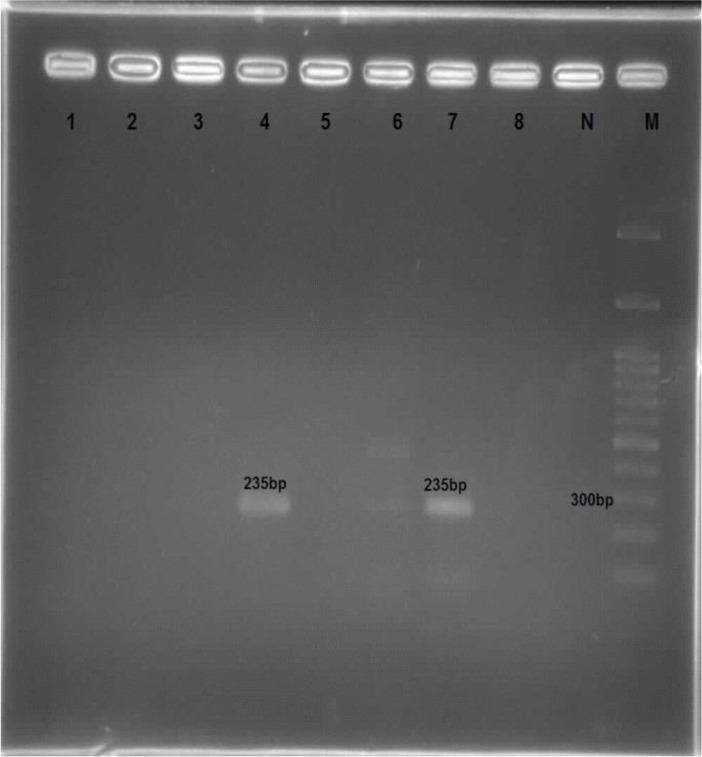
Agarose gel electrophoresis of Semi Nested PCR products using specific primers TBR2 and TL4 (235bp) M 100bp ladder DNA marker; N negative control. *Theileria lestoquardi* (lane 4, 7: 235bp)

**Fig. 8.**
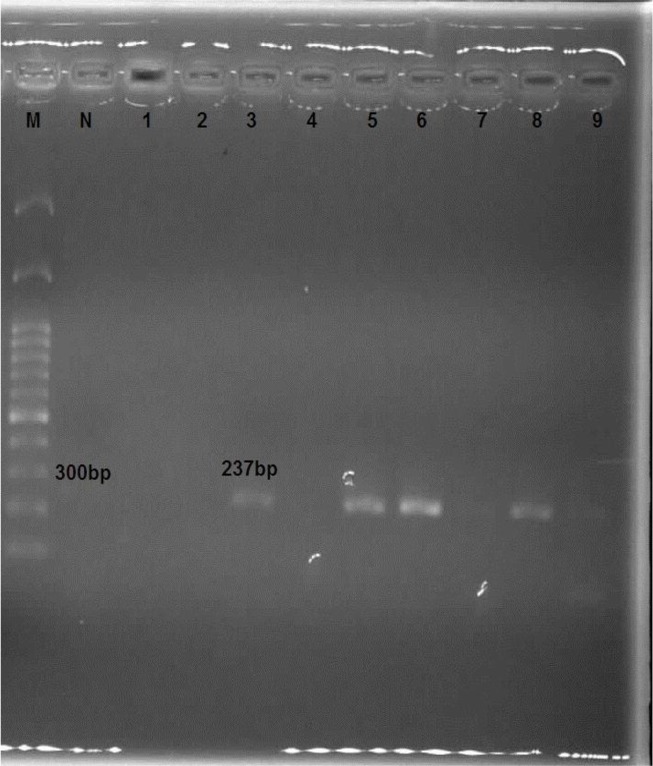
Agarose gel electrophoresis of Semi Nested PCR products using specific primers TBR2 and TO3 (237bp) M 100bp ladder DNA marker; N negative control. *Theileria ovis* (lane 3, 5, 6, 8: 237bp)

The Semi-nested PCR was done with the same reaction in the first round. The PCR products were also electrophoresed through a 1.5% agarose gel to assess the presence of a special band of *T. lestoquardi* (235bp) and *T. ovis* (237bp) (Fig. 7, 8). Negative control (no template) was always run simultaneously with our PCR experiments. PCR product was analyzed on 1.5% agarose gel in 0.5 XTBE buffer and visualized using ethidium bromide and an UV illuminator.

## Results

Overall, 110 ticks (78 males and 32 females) were collected and generally two genera and six species including *Rh. bursa* (9.1%), *Rh. sanguineus* (29.1%), *Rh. turanicus* (10.9 %), *Hy. asiaticum asiaticum* (23.63%), *Hy. excavatum* (10.9%), *Hy. anatolicum* (16.37%) were detected.

After removing the salivary glands of the ticks, genomic DNA of each tick was extracted using a DNA extraction kit (MBST, Iran) according to the kit instruction, and then the infection was evaluated by PCR method. Eighteen ticks including 9 *Hy. asiaticum asiaticum*, 5 *Rh. turanicus*, and 4 *Rh. sanguineus*, were infected with *Theileria*. None of the *Rh. bursa*, *Hy. excavatum* and *Hy. anatolicum* was not infected with *Theileria*. The analysis of PCR product using TBF2 and TBR2 primer showed that of 80 blood samples obtained from sheep, 50 cases (62.5%) were positive (Fig. 6). The results of the Semi-Nested PCR assays on blood sample determined 29 (67.45%) and 14 (32.55%) cases were infected by *T. ovis* and *T. lestoquardi*, respectively. The most infection in Zabol is related to *T. ovis*, and this species is common in this region (Fig. 7, 8).

## Discussion

Molecular examination of 80 sheep blood samples showed that 50 sheep (62.5%) were infected with *Theileria. Theileria ovis* infection was 67.45% and *T. lestoquardi* 32.55% respectively. Moreover, no co-infection was detected. According to our results, the prevalence infection with *T. ovis* species is more than *T. lestoquardi* in zabol. In this study, of 110 ticks collected from different areas of Zabol, the most common ones in the regions were *Rh. sanguineus* with 29.1%, followed by *Hy. asiaticum asiaticum* with 23.63%, *Hy. anatolicum* with 16.37%, *Rh. turanicus* and *Hy. excavatum* with 10.9% and finally *Rh. bursa* with 9.1%.

After evaluating ticks infection by PCR method, three species of *Rh. turanicus*, *Rh. sanguineus* and *Hy. asiaticum asiaticum*, were infected. Considering the role of these tick species in the transmission of *Theileria*, important measures for eradication of them should be considered. In Fars and Kazeroun (12), 100 blood and tick samples were collected to identify the species of *Theileria* and tick vector. Overall, 46% of blood smears from sheep infected with *Theileria*, while 76% of blood samples analyzed by Semi-nested PCR were infected with this parasite. The prevalence of *T. ovis* was 43%, *T. lestoquardi* 3% and 30% mixed infection. The result is also the same as our findings because in their study the prevalence of *T. ovis* was higher than *T. lestoquardi*. In contrast to our study, simultaneous infections of these two species were found in their study. The most common tick species were *Rh. turanicus* (48.8%), *Hy. anatolicum anatolicum* (42.2%), and *Hy. Marginatum* (8.8 %), respectively. In southern Khorasan Razavi (13) on 150 sheep blood samples, infection with *Theileria* species was reported in 18.6% of blood smears. Using Semi-nested PCR, *T. ovis* infection was 58.6% and *T. lestoquardi* 6.6%. The result of this study also showed that the prevalence of *T. ovis* is much higher than *T. lestoquardi*. *Rhipicephalus turanicus* was positive for *Theileria* infection. In Lorestan Province, 219 ticks were collected from the sheep’s body, and after the PCR test, *Rh. sanguineus*, *Hy. anatolicum anatolicum* were infected with *Theileria* species (14).

*Hyalomma detritum* was identified as vector of *T. lestoquardi* in Lorestan Province, as well as *Hy. anatolicum anatolicum* and *Rhipicephalus* in Fars Province (15). In a study in the eastern and southeastern parts of Iran using nested-PCR (16) reported 56% infection with *Theileria*, of these the infection rate was reported by *T. ovis* 12.5% and *T. lestoquardi* 87.5%. Infection with *T. lestoquardi* was reported to be more than *T. ovis*, which is not consistent with the results of the present study. In a study to detect *Theileria* and *Babesia* species in sheep of Ahwaz using PCR-RLFP method (17), 119 blood samples were collected and the following results were obtained: 89% (106/119) of the total samples were positive for *Theileria* infection. 91.5% (97/96%) of *Theileria* positive samples were identified as *T. ovis* and 83.2% (106/3) were *T. lestoquardi*. In the molecular study of malignant ovine Theileriosis in Oman observed the nucleotide sequence of PCR product consistent with *T. lestoquardi* (18). In Turkey, 2241 Ixodid ticks were isolated from sheep and goats, of which 14.86% were infected by *Theileria* and *Babesia*. The infection levels of each species were as follows: 0.691% *Haemaphysalis parva*, 1.47% *Rh. sanguineus*, 1.84 *Ixodes Ricinus*, 2.86% *Rh. turanicus*, 5.57% *Hy. marginatum*, 6.2% *Rh. Bursa* (19).

## Conclusion

*Theileria ovis* has a high prevalence among the sheep of zabol and *Hy. asiaticum asiaticum*, *Rh. sanguineus* and *Rh. turanicus* may be the main vectors of *Theileria* species in this area.
